# Nanoparticle exposure driven circulating bioactive peptidome causes systemic inflammation and vascular dysfunction

**DOI:** 10.1186/s12989-019-0304-6

**Published:** 2019-05-29

**Authors:** Ekaterina Mostovenko, Tamara Young, Pretal P. Muldoon, Lindsey Bishop, Christopher G. Canal, Aleksandar Vucetic, Patti C. Zeidler-Erdely, Aaron Erdely, Matthew J. Campen, Andrew K. Ottens

**Affiliations:** 1 Department of Anatomy and Neurobiology, Virginia Commonwealth University, Box 980709, Richmond, VA 23298-0709 USA; 2 Department of Pharmaceutical Sciences, University of New Mexico, Albuquerque, NM 87131 USA; 3 Pathology and Physiology Research Branch, National Institute for Occupational Safety and Health, Morgantown, WV 26505 USA

**Keywords:** MWCNT, Nanomaterial, Lung, Peptidome, MMP, Mass spectrometry, Carbon nanotubes, Inflammation, Vascular dysfunction, Thrombospondin, Nanoparticles

## Abstract

**Background:**

The mechanisms driving systemic effects consequent pulmonary nanoparticle exposure remain unclear. Recent work has established the existence of an indirect process by which factors released from the lung into the circulation promote systemic inflammation and cellular dysfunction, particularly on the vasculature. However, the composition of circulating contributing factors and how they are produced remains unknown. Evidence suggests matrix protease involvement; thus, here we used a well-characterized multi-walled carbon nanotube (MWCNT) oropharyngeal aspiration model with known vascular effects to assess the distinct contribution of nanoparticle-induced peptide fragments in driving systemic pathobiology.

**Results:**

Data-independent mass spectrometry enabled the unbiased quantitative characterization of 841 significant MWCNT-responses within an enriched peptide fraction, with 567 of these factors demonstrating significant correlation across animal-paired bronchoalveolar lavage and serum biofluids. A database search curated for known matrix protease substrates and predicted signaling motifs enabled identification of 73 MWCNT-responsive peptides, which were significantly associated with an abnormal cardiovascular phenotype, extracellular matrix organization, immune-inflammatory processes, cell receptor signaling, and a MWCNT-altered serum exosome population. Production of a diverse peptidomic response was supported by a wide number of upregulated matrix and lysosomal proteases in the lung after MWCNT exposure. The peptide fraction was then found bioactive, producing endothelial cell inflammation and vascular dysfunction ex vivo akin to that induced with whole serum. Results implicate receptor ligand functionality in driving systemic effects, exemplified by an identified 59-mer thrombospondin fragment, replete with CD36 modulatory motifs, that when synthesized produced an anti-angiogenic response in vitro matching that of the peptide fraction. Other identified peptides point to integrin ligand functionality and more broadly to a diversity of receptor-mediated bioactivity induced by the peptidomic response to nanoparticle exposure.

**Conclusion:**

The present study demonstrates that pulmonary-sequestered nanoparticles, such as multi-walled carbon nanotubes, acutely upregulate a diverse profile of matrix proteases, and induce a complex peptidomic response across lung and blood compartments. The serum peptide fraction, having cell-surface receptor ligand properties, conveys peripheral bioactivity in promoting endothelial cell inflammation, vasodilatory dysfunction and inhibiting angiogenesis. Results here establish peptide fragments as indirect, non-cytokine mediators and putative biomarkers of systemic health outcomes from nanoparticle exposure.

## Background

Ultrafine (< 0.1 μm) particulate represents a unique toxicological burden with the ability to circumvent or frustrate normal mucociliary clearance, deposit deep into alveolar regions, and produce prolonged retention and enhanced toxicity [1–3]. Yet the health implications of environmental or occupational nanoscale particulate extend well beyond the lung, with inflammation and dysfunction evident after exposure in the cardiovasculature and other organs like the brain [4–7]. Among engineered nanoparticles, multi-walled carbon nanotubes (MWCNT) stand out as particularly hazardous given their durability and fibrous shape akin to asbestos [8]. Systemic inflammation [9], atherosclerosis [10], vascular dysfunction [11], and even cognitive deficits [12] have been reported from MWCNT pulmonary exposure, impacting other organ systems such as the liver, kidneys [13] and brain [14]. Particle translocation is one possible explanation for the extensive systemic response to nanoscale particulate [13, 15, 16]. However, data on translocation from the lung can be ambiguous for insoluble materials like MWCNT, with several studies showing no evidence for it [17, 18]. With detailed tracking of MWCNT following oropharyngeal aspiration, Mercer et al. showed only a modest 1% clearance to the lymphatics within 24 h, with less than 0.01% MWCNT transferred to other organs [19]. Implicated is an indirect mechanism underlying acute systemic effects that may extend to other partly soluble materials. Brook et al. proposed that proinflammatory mediators travel from the lung into circulation, the so-called “systemic spill-over” mechanism [6]. Yet cardiovascular deficits from nanoparticle exposure have been reported without pulmonary inflammation [20, 21]. This suggests that extra-pulmonary outcomes are mediated by as-yet-unknown, non-cytokine, bioactive molecules released into the circulation [22, 23].

Cardiovascular deficits are among the earliest and widest reported systemic effects of nanoparticle exposure [10, 24]. Work from our group and others demonstrates impaired endothelium-dependent relaxation when naïve aortic vessels are treated with serum from MWCNT-exposed animals [25, 26]. Yet, vessels from CD36 knockout animals were largely unaffected by serum from wild-type mice exposed to MWCNT [25]. In reverse, vasodilatory deficits in naïve aortas were largely muted when incubated with serum from thrombospondin (TSP) knockout animals exposed to MWCNT [26], pointing to a TSP-CD36 mediated effect. However, this does not explain how treatment with serum from MWCNT-exposed matrix metalloproteinase 9 (MMP9)-null mice in part rescues vasodilation in naïve vessels [25]. The latter result speaks to the broader relevance of matrix protease activation within the lung and its potential involvement in transducing systemic effects after pulmonary insult [27, 28]. Thus, we posited that protease-generated peptide fragments represented an untested bioactive fraction in promoting systemic pathobiology. MWCNT-induced lung responses may activate a diverse set of matrix proteases to foster the release of peptide products across the lung-blood barrier, which may mediate at least part of the extra-pulmonary burden. Recent advances in data-independent quantitative mass spectrometry provides an ideal platform to assess endogenous unknown products [29]. Here, we used unbiased mass spectrometry to analyze paired bronchoalveolar lavage and serum biofluids to characterize Mitsui-7 MWCNT-aspiration induced peptidomic fragments introduced into circulation 4 h after insult with relevance to ongoing lung pathobiology. The bioactive contribution of the serum peptide fraction was functionally interrogated ex vivo, with results supporting a newly proposed peptide-mediated indirect mechanism for the peripheral effects of nanoparticle lung exposure.

## Methods

### Animal model and sample collection

Specific pathogen-free male C57BL/6 J mice (Jackson Laboratory) were housed in an Association for Assessment and Accreditation of Lab Animal Care International approved animal facility at the National Institute for Occupational Safety and Health. Animal care and use procedures were conducted in accordance with the “Public Health Service Policy on Humane Care and Use of Laboratory Animals” and the “Guide for the Care and Use of Laboratory Animals”. Food and water were provided ad libitum in ventilated cages in a temperature and humidity controlled environment with a 12-h light/dark cycle. Eight-week-old C57BL/6 J mice, were exposed to MWCNT (Mitsui-7, Hodogaya, Japan, > 99% carbon purity) via oropharyngeal aspiration at 0 μg, 10 μg, or 40 μg (*n* = 6 per group) [30]. The MWCNT material was found negative for LPS using a ToxinSensor Chromagenic LAL Assay Kit (GenScript). MWCNT was prepared in dispersion media (DM) consisting of mouse serum albumin (0.6 mg/mL) and 1,2-dipalmitoyl-sn-glycero-3-phosphocholine (10 μg/mL). The average MWCNT was 49 nm in diameter and 3.86 μm in length (geometric SD = 1.94) [30]. Matched serum and bronchoalveolar lavage fluid (BALF) were collected 4 h following aspiration under anesthesia as previously described [23]. The 4 h collection time was selected here to remain consistent with prior published work using this model that demonstrated in vivo *and* ex vivo vascular outcomes of MWCNT exposure [14, 23, 25].

### Endogenous peptide enrichment and mass spectrometry

Matched serum and BALF were processed via the same protocol with proportional adjustment for their different starting volumes of 40 μl for serum and 120 μl for BALF given pilot results showing a 3–4 fold difference in peptide concentration. Biofluids were clarified by centrifugation through a 0.22 μm Ultrafree-MC filtration unit (EMDMillipore, Billerica, MA) using manufacturer instructions. Samples were then denatured for 30 min at room temperature (18 mM TCEP final concentration) in presence of HALT inhibitor cocktail (Thermo Scientific, Rockford, IL) and 20% final concentration acetonitrile. Reduced thiols were acetylated with iodoacetamide at a final concentration 30 mM with a 30 min incubation in the dark at room temperature. Samples were transferred onto pre-cleaned MicroCon YM-30 centrifugal filter units (EMDMillipore) and centrifuged per manufacturer instructions to isolate endogenous peptides from proteins and vesicles. The retentate was acidified using 0.4% formic acid to further disrupt peptide binding with collection via a second centrifugation of the filter unit. Resultant peptide-enriched filtrates were loaded (4.5 μl) onto a Symmetry C18 reversed-phase column to remove lipids, reagents and salts. The peptidomic fraction for each serum sample was separated using a NanoAcquity UPLC (Waters, Milford, Massachusetts) online with a Waters Synapt G2 tandem mass spectrometer as described previously [31]. Briefly, the peptide fraction was separated on a 150 mm × 75 μm HSS T3 reversed-phase capillary column at 55 °C for 65 min with an elution gradient from 6 to 44% acetonitrile in water (0.1% formic-acid modified). The Synapt G2 was operated with ion mobility enabled data-independent acquisition (UDMSe) at a nominal 25,000 resolving power [32]. The precursor mass range was optimized between 400 and 1800 m/z to account for larger endogenous peptides.

### Mass spectral data processing and analysis

Spectra processing was performed employing PLGS v3.0.2 software (Waters) as described previously [31]. Ion tables for matched BALF and serum samples were clustered together in matching retention time (±2 min), drift time (±4 bins), and ion mass (±12 ppm) with EndogeSeq. Results were filtered to include only reproducible ion events observed in two-thirds or more of the biological replicates. For ions categorically falling below the limit of detection across all replicates in a group, a randomly generated set of values was imputed with a mean and coefficient of variance equating the limit of quantification observed across that group’s replicates [33]. The clustered ion matrix was then median centered and log_2_ transformed. Fold changes were calculated relative to the mean for the DM (0 μg MWCNT) vehicle control group. Ions found significantly responsive to MWCNT treatment in serum and BALF biofluids were assessed to identify an overlap with known MMP and ADAM/TS substrates using the MEROPS database [34] and with proteins with predicted secretory domains using the SignalP algorithm [35]. The search workflow included no enzyme specificity for assessing endogenous measures with precursor and product ion match limits of 6 and 12 ppm, respectively. A random-decoy database method was used to control false peptide identification to under a 10% false discovery rate (FDR) using the peptide score, which is highly dependable given the high-resolution tandem mass spectral measures [36]. Matched product ion spectra were visualized using mMass software [37]. Identified peptides were further characterized using the enrichment analysis tools in ToppGene [38] and STRING [39] online software suites, with results adjusted to a 5% FDR.

### Matrix protease expression analysis in the lung

Four hours after DM vehicle, 10 μg or 40 μg MWCNT oropharyngeal aspiration, mice were euthanized and their left lung lobe was ligated while bronchoalveolar lavage was performed on the right lung lobe. BALF and whole lung homogenates were assessed for MMP9 protein levels using an enzyme linked immunosorbent assays according to manufacturer’s instructions (Boster, Pleasanton, CA).

Broader matrix protease and tissue inhibitors of metalloproteinases were assessed in an existing lung tissue microarray dataset. Animals were exposed for 4 h to Mitsui-7 MWCNT by inhalation to deposit approximately 4 μg or 40 μg as previously described [40]. Lung tissue was assessed for gene expression on an Illumina platform as previously described [40]. In summary, 375 ng of RNA was used to generate cRNA for hybridization to the arrays. MouseRef-8 BeadChips were analyzed on an Illumina BeadStation 500G reader (Illumina, San Diego, CA). Data processing and differential expression analysis was performed using the Limma R package as described elsewhere [41].

### Serum cumulative inflammatory potential assay

Mouse endothelial cells were obtained from a commercial vendor (Cell Biologics, Chicago, IL) and maintained according to manufacturer’s recommendations at 37 °C and 5% CO_2_ with complete endothelial cell medium supplemented with 6% fetal bovine serum. All experiments were performed between passages 3 and 8. Assays were batched by exposure to enhance consistency and comparability across samples. Obtained endothelial cells were treated 4 h in vitro with the enriched peptide fraction from MWCNT exposed or DM control mouse serum to assess the serum cumulative inflammatory potential as previously published [42]. In short, endothelial cells were serum starved overnight and incubated with culture media supplemented at a final concentration of 5% subject peptide fraction for 4 h and harvested. RNA was isolated using the RNeasy Mini Kit (QIAGEN, Germantown, MD) and reverse transcribed prior to quantitative real-time PCR. Expression of interleukin 6 (*Il6*), C-C motif chemokine ligand 2 (*Ccl2*), C-C motif chemokine ligand 5 (*Ccl5*), vascular cell adhesion molecule 1 (*Vcam1*), (*Icam1*), tumor necrosis factor alpha (*Tnfa*), and transforming growth factor beta (*Tgfb*) (Applied Biosystems, Foster City, CA) were measured using the TaqmanR Gene Expression protocol (ThermoScientific, Waltham, MA) following the manufacturer’s instructions. Relative gene expression normalized to the endogenous control TATA-Box Binding Protein gene was analyzed using the 2^−ΔΔCT^ method.

### Myography vascular function assay

Analysis was performed as described previously [25]. Briefly, 2 mm segments of thoracic aorta rings were isolated from naïve C57BL/6 J mice, cleaned of connective tissue and mounted in a 4-chamber multi-wire myograph (610 M; Danish Myo Technology A/S, Aarhus, Denmark) submerged within solutions continuously maintained at 37 °C and bubbled with 21% O_2_–5% CO_2_ in nitrogen. Naïve vessels were initially submerged in physiological saline solution (PSS): 4.7 mM KCl, 119.0 mM NaCl, 25.0 mM NaHCO_3_, 5.5 mM glucose, 1.2 mM MgSO_4_, 1.2 mM KH_2_PO_4_, 0.025 mM EDTA, 2.5 mM CaCl_2_ and equilibrated for 30 min. Vessel viability was then tested by assessing a contractile response to a high-concentration potassium chloride physiological saline solution (KPSS): 58.9 mM KCl, 64.9 mM NaCl, 25.0 mM NaHCO_3_, 5.5 mM glucose, 1.2 mM MgSO_4_, 1.2 mM KH_2_PO_4_, 0.025 mM EDTA, 2.5 mM CaCl_2_. Afterwards, all vessels were washed four times with PSS and equilibrated for 30 min. The naïve vessels were then treated and allowed to stabilize ex vivo in PSS spiked at 1% (v/v) with the serum peptide fraction derived from the animals previously exposed to MWCNT or DM in vivo. Then the cumulative concentration-response curve to acetylcholine (10^− 9^ to 10^− 4^ M) was acquired using LabChart software.

### Electrical wound-healing angiogenesis assay

Mouse endothelial cells were plated at 2 × 10^5^ cells/mL and grown to confluence on a 96-well 96W1E+ electrode plate coated with 0.01% poly-L-lysine for an Electric Cell-Substrate Impedance System (ECIS; Applied Biophysics, Troy, NY). Transmembrane resistive impedance was recorded continuously (4 Hz) until establishment of tight intracellular contact. Synthetic TSP_402–460_ peptide or the serum peptide fraction derived from the animals previously exposed to MWCNT or DM in vivo were added to each well at 5% (v/v) in media. After a 4-h in vitro treatment, a 1 mA current pulse was passed through a central electrode on each well to instigate a wound. Angiogenesis regrowth towards the center of the well was then monitored as the recovery of transmembrane impedance. The ECIS assay was preferred to the physical scratch method, as the protein coating on the wells is unperturbed by the electrical stimulus. Results were reported as the average normalized impedance per hour as a percent of baseline impedance from the hour prior to treatment.

### Exosome characterization

Serum exosomes were purified first by passing through a 0.22 μm Ultrafree MC centrifugal unit to eliminated larger-sized debris and apoptotic bodies and then by size-exclusion chromatography to resolve from lipovesicles and serum protein using a 5 mm × 10 cm column packed with Sephacryl S-500 HR media (GE Bio-Sciences, Uppsala, Sweden) at 0.2 mL/min in PBS buffer. The optimized exosome fraction was collected from 3 to 5 min and assessed using a Zetasizer Nano S90 (Malvern Panalytical, Malvern, UK). Exosome samples were also fixed in 4% paraformaldehyde in 0.15 M sodium cacodylate buffer 1:1 (v:v), a 10 μl drop was placed onto parafilm and covered with a formvar-coated grid. Mounted samples were rinsed with PBS and fixed again with 1% glutaraldehyde in 0.1 M sodium cacodylate. The sample was then rinsed and negative stained using 0.5% aqueous uranyl acetate and air dried overnight. A JEOL JEM-1230 transmission electron microscope with Gatan Orius SC1000 side-mount CCD camera was used for imaging.

### Gene expression analysis examined for c-Jun regulatory targets

RNA was extracted from naïve endothelial cells, as described earlier for the serum cumulative inflammatory potential assay, after a 4-h in vitro treatment with serum collected 4-h after exposing mice in vivo to DM vehicle, 10 μg, and 40 μg MWCNT. Extracted RNA was analyzed using Affymetrix GeneChip Mouse 430 2.0 arrays according to the manufacturer’s protocol and as described previously [14]. Briefly, 1 μg of total RNA was used to produce biotinylated cRNA, synthesized by in vitro transcription using the GeneChip IVT Labeling Kit (Affymetrix) and fragmented and hybridized to the Affymetrix GeneChip following the chip protocol. Arrays were scanned with an Affymetrix GeneChip Scanner 3000. Results meeting a minimal log change ratio of 0.5 from DM control and *p* < 0.01 were then selected if known as c-Jun regulatory targets.

### Statistical analysis

Statistical analysis of log_2_-transformed mass spectral peptide data was performed in MultiExperimentViewer v4.9 [43] using the one-way ANOVA function and the significance level corrected for multiple peptide measures to a 5% FDR using the Benjamini-Hochberg method [44]. Statistically responsive peptides were assessed for intensity correlation across matched serum and BALF samples using linear regression analysis in SigmaPlot v13. MMP9 ELISA and vesicular size results were assessed using one-way ANOVA with Holm-Sidak post hoc or one-way ANOVA on Ranks (for results with unequal variance) with Dunnett’s post hoc testing for multiple comparisons in SigmaPlot. Serum cumulative inflammatory potential assay results were assessed by two-way ANOVA with dose and denaturing as factors and Holm-Sidak post hoc testing in SigmaPlot. Myography and ECIS results were assessed by two-way ANOVA with dose as the first factor and acetylcholine concentration (myography) or time (ECIS) as the second factor followed by Holm-Sidak post hoc testing in SigmaPlot. Microarray results were analyzed using the Limma R package in Bioconductor v3.8, which uses linear regression models for each gene assessed for the Bayes moderated t-statistic and associated *p*-values, corrected for multiple testing using the Benjamini-Hockberg method. Data were plotted as the mean ± SE, with n denoting the number of biological replicates described per figure legend.

## Results

### MWCNT-induced peptides overlap between the lung and circulation

**Fig. 1:**
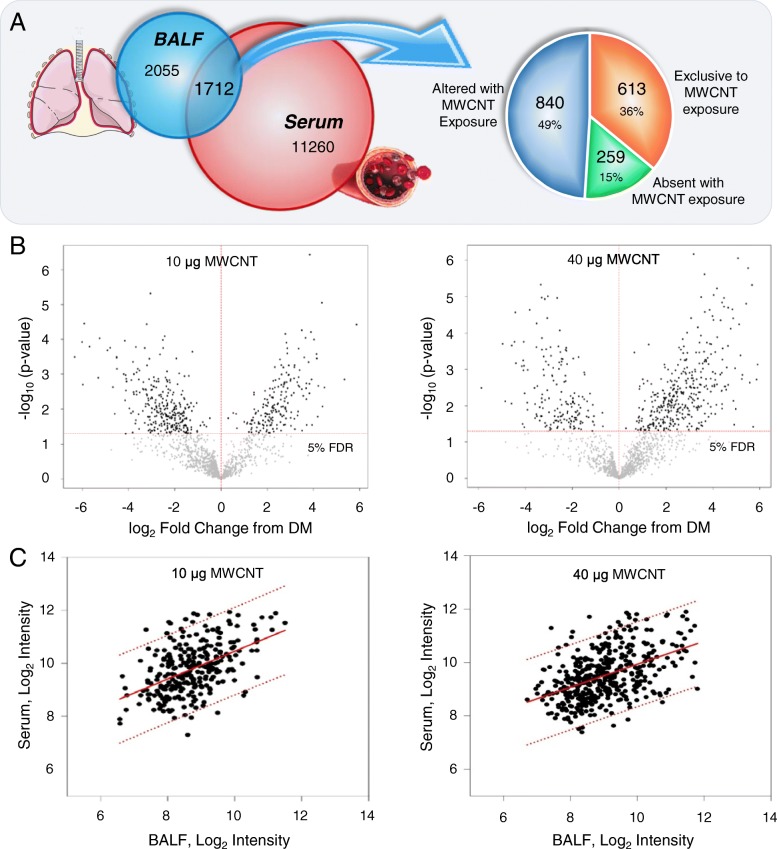
MWCNT-responsive peptidome exhibited correlatory abundance between BALF and serum. The complex molecular response 4-h after MWCNT exposure within enriched-peptide fractions was assessed by mass spectrometry in animal-paired BALF and serum specimens. **a** Overlapping factors (1712) included 613 measures detected exclusively or 259 lost entirely from fluids in response to MWCNT exposure relative to DM control treatment with the remainder found modulated by exposure. **b** Volcano plots depicting the fold-change and significance level between MWCNT exposed and DM control animals for measures from the enriched-peptide fraction found across BALF and serum, *p* adjusted to a 5% FDR. **c** Linear regression plots of mass spectral intensity measures between animal-paired BALF and serum specimens. Plotted are those peptide measures that reached the 5% FDR significance level. The regression line and 95% confidence interval are show as solid and dashed lines, respectively. All data were generated using matched BALF and serum from *n* = 6 replicate exposures per dose

Enriched-peptide fractions from animal-matched serum and BALF specimens were isolated by size-fractionation and hydrophobic solid-phase extraction from proteinaceous and lipid components and assessed by data-independent mass spectrometry to identify cross-talk between the lung and circulation. As expected, the depth of detection was more limited for BALF (3767 reproducible measures), at 29% of that for serum (12,972 reproducible measures). The lavage procedure involves the rapid partition of the bronchoalveolar surface molecular mass into the lavage buffer for a dilute representation of the airway epithelial lining fluid, which limits recovery and assessment [45]. That said, 1712, or nearly half of all reproducible measures assessed in BALF were detected within serum, demonstrating substantial overlap between the two compartments (Fig. 1a). Interestingly, more than half of the BALF-serum common measures (872 of the 1712) were exclusively detected either among replicate specimens from MWCNT or DM vehicle treatment groups, 613 (70%) of those solely detected after MWCNT exposure (i.e., not in any specimens from DM treated animals). Statistically, 841 of 1712 species were responsive to MWCNT treatment at a 5% FDR (Fig. 1b). Of those, 567 species also demonstrated a significant abundance correlation between BALF and serum: 10 μg exposure, R = 0.49, F = 84.7, *p* < 0.001; 40 μg exposure R = 0.48, F = 126.5, *p* < 0.001 (Fig. 1c). Thus, results here corroborated a robust molecular exchange between the lung and the circulation following MWCNT exposure allowing for further focused study of mediators of circulating systemic bioactivity in examining the identity of BALF-serum common factors as having more direct relevance to MWCNT-induced pulmonary pathobiology.

### MWCNT-responsive peptides identified from matrix protease substrates

**Fig. 2:**
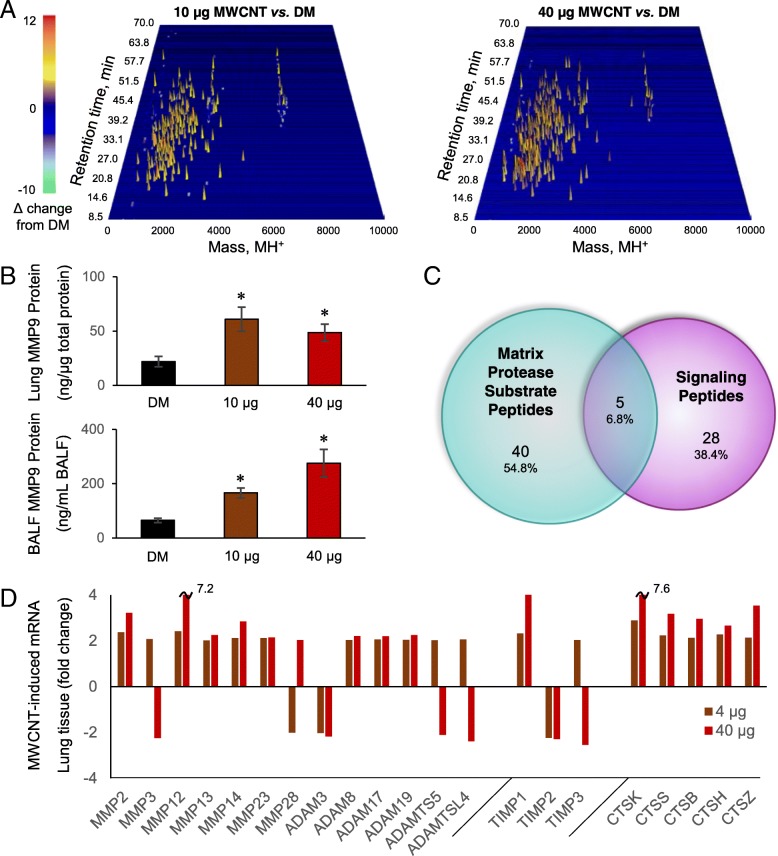
Circulating MWCNT-induced peptides relate to matrix protease activation. **a** Waterfall plots depicting the MWCNT-induced fold-change among responsive factors within the enriched peptide fraction plotted three-dimensionally against reversed-phase retention time and the charge-reduced protonated mass (MH+). **b** MMP9 protein abundance in animal-matched lung tissue and BALF 4-h after MWCNT exposure as assessed by ELISA and presented as the mean ± SE, *n* = 5 replicate mouse exposures per dose, *p < 0.05. **c** Venn diagram of identified MWCNT-responsive peptides in association with databases of matrix protease substrates and predicted secreted signaling motifs. **d** Gene expression changes in lung tissue across matrix proteineases of the MMP, ADAM and ADAMTS families, tissue inhibitors of metalloproteinases (TIMPs) and lysosomally-derived cathepsins (CTS) following a 4-h inhalation exposure to MWCNT. Results reported for proteases that exhibited a two-fold or greater response to MWCNT and reached significance at an FDR of 5% as assessed by Illumina BeadChip and presented at the mean response across *n* = 4 replicate mouse exposures per dose

The fold-change response for the serum enriched-peptide fraction is illustrated across chromatographic and mass dimensions via 3D waterfall plots (Fig. 2a). Evident is a predominant increase in the assessed factors following 10 μg or 40 μg treatment (colored yellow to orange). The mass distribution fell between 1000 and 7000 Da, which was consistent for peptides, and generally exclusive of masses for small molecule metabolites below 1000 Da and proteins above 7000 Da. The chromatographic retention profile reflected complete elution between 10 and 36% acetonitrile, which was confirmatory of an enriched-peptide fraction and effective lipid removal. Figure 1b illustrated a greater number of increased peptide with the 40 μg exposure relative to the 10 μg MWCNT. Figure 2a illustrates that the majority of additional peptides with 40 μg treatment are larger in mass, with greater density around 3 kDa and 6.5 kDa. As larger mass fragments are more likely to include complete binding motifs, they may convey broader bioactivity.

The diversity of the peptidomic response suggests extensive proteolytic processing within the lung and subsequently in the circulation. Previously we demonstrated that MWCNT-induced vasodilatory deficits were dependent on MMP9 [25]. An MMP9 elevation in lung (F = 5.9, *p* = 0.013) and BALF (H = 11.6, *p* = 0.003) support its potential involvement in peptide fragment generation (Fig. 2b). Yet MMP9 cleavage alone is insufficient to explain the shear multiplicity of peptides produced in response to MWCNT exposure. We thus looked farther afield in identifying peptides related to known substrates broadly across matrix proteases, including MMPs, ADAMs, and ADAMTSs. Resources available from the MEROPS peptidase project of the European Bioinformatics Institute provided a concise source of known substrates for these proteases that could then be used to significantly reduce the sequence search space [34], as beneficial to identifying endogenous peptide mass spectra [46]. Metalloproteases are further associated with extracellular signal-peptide release [47]. The SignalP resource from the Technical University of Denmark Bioinformatics unit extended our search capability to included predicted peptides of excreted proteins [35]. From these resources, 73 of the BALF-serum common measures found statistically responsive to MWCNT at one or both doses (10 μg or 40 μg) were identified (Fig. 2c and Table 1), relating the genesis of MWCNT induced peptides to known matrix protease substrates and secreted proteins. To substantiate further involvement of additional proteases, we mined an existing lung tissue microarray dataset exhibiting the effects of inhaled Mitsui-7 MWCNT with four-hour deposited doses on par with those modeled here. Array analysis in lung tissue showed significant upregulation for a broad array of other MMPs, ADAMs and ADAMTSs with MWCNT exposure (Fig. 2d). Furthermore, tissue inhibitors of metalloproteinases (TIMPs) were likewise modulated, with TIMP1 significantly increased at both doses, TIMP2 significantly decreased at both doses, and TIMP3 showing an opposing response between doses. Other proteases are also known to influence remodeling of the ECM after MWCNT exposure, to include lysosomal-released cathepsins [48]. Microarray data showed significant increased at both doses for cathepsins B, H, K, S and Z. Taken together, these data support involvement of multiple proteases, with likely promiscuous activation of one by another and tandem cleavage of common substrates, in generating the peptidomic response observed here.

**Table 1:** Identified sequences within the MWCNT-induced peptidome

Peptide_Sequence	Parent_Protein_Name	Symbol	Accession	Homo-logue	Source	Peptide Mass	Precursor Mass	Prec. RT^a^	Prec. DT^b^	Prec. Merr^c^	Prod. Merr^d^	Peptide Score	FDR
QDALSGSSDLLELLLQEDSRSGTGSAASGSLGSGLGSGSGSGSHEGGSTSASLTRSSQSSHTSKYFGSLD	Period circadian protein homolog 1	Per1	O35973		MEROPS	6731.1214	6750.1381	40.42	54.58	0.25	2.57	7.679	0.000
SFDQVDPVQSTFPTETGGLST	von Willebrand factor D and EGF domain-containing protein	Vwde	A0A0N4SVZ4		SignalP	2194.0066	2213.0318	39.54	46.69	3.11	3.28	7.510	0.000
TERFGQGGAGPVGGQGPRGMGPGT	Splicing factor proline- and glutamine-rich	Sfpq	Q8VIJ6		MEROPS	2209.0446	2228.0428	39.21	47.91	9.16	3.65	7.448	0.000
RYTQKAPQVSTPTLVEAARNLGRVGTKC	Serum albumin	Alb	P07724		MEROPS, SignalP	3082.6458	3101.6650	29.18	51.67	0.27	5.34	7.275	0.000
GRPERQFFVKWQGMSYWHCSWVSELQ	Chromodomain-helicase-DNA-binding protein 4	Chd4	Q6PDQ2		MEROPS	3309.5389	3328.5559	34.18	52.67	0.42	5.62	7.256	0.000
KDTCFSTEGPNLVTRCKD	Serum albumin	Alb	P07724		MEROPS, SignalP	2108.9619	2127.9844	18.90	45.86	1.94	5.90	7.239	0.000
CKAADKDTCFSTEGPNLVTRCKD	Serum albumin	Alb	P07724		MEROPS, SignalP	2654.1887	2673.2104	18.12	53.79	1.25	5.91	7.238	0.000
PAGANGEKGEVGPPGPSGSTGARGAPGERGETGPPGPAGFAGPPGADGQPGAKGDQGEAGQKGDAGAPGPQGPSGA	Collagen alpha-1(II) chain	Col2a1	P28481		MEROPS, SignalP	6659.1069	6678.1019	36.86	52.51	3.51	5.98	7.234	0.000
NKGPVKVVVGKTF	Protein disulfide-isomerase A4	Pdia4	P08003		MEROPS	1353.8132	1372.8346	32.85	63.44	2.20	6.41	7.212	0.000
PPSRSNSEQPGSLHSSQGLQMGPVEESWFSSSPEYPQDENDRSVQAKLQEEASYHAFGL	Receptor-interacting serine/threonine-protein kinase 1	Ripk1	Q60855		MEROPS	6499.9588	6518.9949	38.98	52.98	2.73	6.81	7.194	0.000
KCLREMYTTHEDVEVGRCVRRFAGVQCVWSYEMQQLFYENYEQNKKGYLRDL	Chondroitin sulfate synthase 1	Chsy1	Q6ZQ11		SignalP	6563.0918	6582.0918	36.11	52.00	2.81	7.39	7.171	0.000
KDRGHREEGEDFSREYGHRVQDHRYPG	Histidine rich calcium binding protein isoform CRA_a	Hrc	G5E8J6		SignalP	3293.5211	3312.5554	44.06	52.77	4.82	7.40	7.170	0.000
VCTAVEDTRPVMDRNTDGQDGAYAEGTTKWPAEENRPQGKPSTKKSQSSKGQEGESCLRT	Dickkopf-related protein 4	Dkk4	Q8VEJ3		SignalP	6607.0786	6626.1046	40.69	57.90	1.15	7.76	7.158	0.000
MNLEEKPAPAA	Apolipoprotein A-II	Apoa2	P09813		SignalP	1151.5645	1170.5826	17.71	46.99	0.23	7.87	7.154	0.000
TMNNEKYPVNLSETRLGWNSFNCSLSKNSNKKDHFTFNNTLEWTARNNFDMVLSE	Protein Vmn2r13	Vmn2r13	L7N1X2		SignalP	6524.0437	6543.0484	37.20	52.69	2.10	8.26	7.142	0.000
TSVPKRRRPSGNGGFLGDPYCSESPQESSCEDGEGSSVMSARQRSAAESSKLSCSDVPDLVR	DNA repair protein complementing XP-G cells homolog	Ercc5	P35689		MEROPS	6685.0674	6704.1050	37.74	53.39	2.88	8.50	7.135	0.000
KPSGLVMARKLLHLELKPAL	Dual specificity mitogen-activated protein kinase kinase 1	Map2k1	P31938		MEROPS	2195.3340	2214.3732	41.79	47.98	9.48	9.58	7.109	0.000
YYVDSEGNRLSGTAFSVGSGSVYAYGVMDRGYSYDLKVEEAYDLARRALYQATYRDAYSG	Proteasome subunit beta type-5	Psmb5	O55234		MEROPS	6677.1145	6696.1121	38.75	53.55	3.12	9.67	7.107	0.000
DLKLCDFGLARVADPDHDH	Mitogen-activated protein kinase 1	Mapk1	P63085		MEROPS	2175.0167	2194.0272	37.28	47.00	3.62	10.35	7.093	0.000
GKEAYAEYHFRVGSEAEGYALQVSSY	Fibrinogen alpha chain	Fga	E9PV24		MEROPS	2892.3354	2911.3501	26.92	50.14	1.29	10.95	7.083	0.000
SLCDLPVHSNKEWSQHLNGASHSRRCQLLLELYPEWNPDNDTGHTMGDPFMLQQSTN	Matrin-3	Matr3	Q8K310		MEROPS	6642.0386	6661.0724	41.86	53.01	2.32	11.24	7.078	0.000
ELFQREVSSVELFSYA	UDP-glucuronosyltransferase	Ugt1a5	B2RT14		SignalP	1884.9258	1903.9390	30.24	88.84	2.73	11.73	7.071	0.000
RPCPTEQLSPSHPPLATCFGSDVDLQLEMAVPQPGQYVLVVEYVGEDSHQEMGVAVHTPQ	Laminin subunit alpha-5	Lama5	Q61001		MEROPS	6608.1184	6627.1148	39.56	58.20	3.33	12.10	7.065	0.000
YLPSGQQLYMSKEM	MCG3425	Vmn2r75	G5E8Z7		SignalP	1655.7688	1674.7799	39.43	53.63	4.38	12.62	7.058	0.000
SFDWLMEQKFDMTFSENSHNLYNAVHALAHALHEMNLQQADNQALGNGKGASSHCLKVNS	Protein Gm10302	Vmn2r47	K7 N709	G3UYU1,K7N5W1	SignalP	6737.1121	6756.1282	40.75	54.32	0.34	12.95	7.054	0.000
AMDVQLHSPAFQFPDVDFLREGEDDRTVCKELRRNSTGCLKMKGQCEKCQELLSVDCS	Clusterin	Clu	Q06890		MEROPS	6871.1801	6890.2217	39.42	55.38	3.38	13.42	7.049	0.000
KLQCENVQDMPVFG	Disintegrin and metalloproteinase domain-containing protein 9	Adam9	Q61072	E9Q638	SignalP	1645.7592	1664.7772	34.40	53.13	0.26	13.81	7.045	0.000
GPQGQFRAPGPQGQMGPQGPPMHQGGGGPQGFMGPQGPQGPPQGLPRPQDMHGPQGMQRHPGPH	pre-mRNA 3′ end processing protein WDR33	Wdr33	Q8K4P0		MEROPS	6509.0183	6528.0352	36.48	52.40	0.24	11.24	7.044	0.000
KEEEEQRRAEEQMLKERE	Eukaryotic translation initiation factor 3 subunit A	Eif3a	P23116		MEROPS	2328.1128	2347.1170	24.79	49.11	6.10	13.98	7.043	0.000
ELVVQDGVTLLTKDEGPGSSLT	X-ray repair cross-complementing protein 5	Xrcc5	P27641		MEROPS, SignalP	2239.1583	2258.1794	25.42	43.46	1.20	15.06	7.033	0.000
LNELDFYEAFMEEPMTLPDKPNSEEELVSFVEEHRRSTLRKLKPESMYETWEDD	Calsequestrin-1	Casq1	O09165		SignalP	6560.0450	6579.0482	39.19	52.73	2.31	16.35	7.022	0.000
SEFPHKFGSCVPHTTRPRRDNEVDGQDYHFVVSREQMEKDLQDNKFLEAGQFNDNL	Disks large homolog 3	Dlg3	P70175		MEROPS	6645.1002	6664.1136	31.59	55.15	0.76	18.09	7.011	0.000
QQRGRSCDVTSNTCLGPSLQTRTCSLGKCDTRLRQNGGWSHWSPWSSCSVTCGVGNVTR	Thrombospondin-2	Thbs2	Q03350		MEROPS	6737.0870	6756.1282	40.75	54.32	3.38	12.95	6.965	0.000
GKMCFQTLTDDDYKSELR	Ephrin type-B receptor 1	Ephb1	Q8CBF3		MEROPS	2187.9929	2207.0319	38.08	46.90	9.43	16.34	6.879	0.017
GCYYYWNTQTNEVTWELPQYLATQVQGLQHYQPSSVTGTEAAFVVNTDMYTKERTT	Formin-binding protein 4	Fnbp4	Q6ZQ03		MEROPS	6549.0270	6568.0537	38.36	53.02	1.26	14.37	6.879	0.017
VPFDGMWLDMNEPSNFVRGSQQGCPNNELENPPYVPGVVGGLLQAATLCASSHQFLSTHY	Lysosomal alpha-glucosidase	Gaa	P70699		MEROPS	6614.0616	6633.0737	39.13	53.55	0.95	14.82	6.874	0.017
GYAANYCDGECSFPLNAHMNATNHALVQTLVHLMNPEYVPKPCCAPTKLNALSVLYFDD	Bone morphogenetic protein 6	Bmp6	P20722		SignalP	6693.0782	6712.1155	37.19	58.90	2.83	12.66	6.869	0.017
GSGEQYRGSVSKTRKGVQCQHWSSETPHKPQFTPTSAPQAGLEANFCRNPDGDSHGPWC	Hepatocyte growth factor-like protein	Mst1	E0CXN0	P26928	SignalP	6577.9900	6597.0184	41.81	53.73	1.52	9.93	6.851	0.017
SCDSALRAYVKDHYSNGFCTVYAKTLDGQQTLLACLESHQFQPKNFWNGRWRSEW	F-actin-capping protein subunit alpha-1	Capza1	P47753		MEROPS	6607.0861	6626.1144	41.74	52.74	1.49	17.04	6.846	0.017
GNVKMTLGMLWTLL	Alpha-actinin-1	Actn1	Q7TPR4		MEROPS	1557.8411	1576.8527	46.69	71.65	4.37	11.30	6.804	0.017
YPGQAPPGAYPGQAPPSAYPGPTAPGAYPGPTAPGAYPGQPAPGAFPGQPGAPGAYPQCSGGYPAAGPYGV	Galectin-3	Lgals3	P16110		MEROPS	6672.0973	6691.0977	39.09	53.61	2.70	12.38	6.801	0.029
TQAFYRVDLSLDFAEMDSPVHWTVE	Protein Gm572	Gm572	B1ARY8		SignalP	2937.3643	2956.3656	26.01	45.98	5.82	13.96	6.800	0.029
FGSGQSSGLTSVSGETSGLSDLSG	Aggrecan core protein	Acan	Q61282		MEROPS	2197.9975	2217.0301	39.32	46.73	6.47	14.94	6.788	0.029
FKASDLDGDLTATREEFTAF	Reticulocalbin-1	Rcn1	Q05186		MEROPS	2215.0433	2234.0482	38.26	48.00	6.09	13.30	6.770	0.029
LYKLDDPSCPRPECYRSCGSSTPDEFPTDLPGTKGNFKLVRHVSFVDCPGHDLLMATM	Eukaryotic translation initiation factor 2 subunit 3 X-linked	Eif2s3x	Q9Z0N1		MEROPS	6652.0840	6671.0899	38.48	52.88	1.88	13.61	6.767	0.029
VDTSFVEVTPTTFREEEGLGSVELSGFPSGETELSGTSGTVDVSEQSSGALDSSGLTSPTPEFSGL	Aggrecan core protein	Acan	Q61282		MEROPS	6665.0991	6684.0975	40.09	53.32	3.00	9.91	6.733	0.029
AEFQPLVEEPKNLVKTN	Serum albumin	Alb	P07724		MEROPS	1937.0258	1956.0455	30.40	47.82	0.68	13.34	6.662	0.037
ESNTNPTGWEPNEENEDETDKYPSFSGSG	CD44 antigen	Cd44	A2APM2		SignalP	3198.2810	3217.2710	36.64	74.43	8.87	12.00	6.655	0.037
RYNQLYTYGYGSVARYNSYQSFQTPQHPSFLFKDKQLSWSATYLPTMQSCWNYGF	Galectin-3-binding protein	Lgals3bp	Q07797		MEROPS	6656.0848	6675.0796	41.58	53.17	3.54	16.89	6.651	0.037
KWNPETVESPGGVEDSQQCLEVEEGPEREQHQESLRSLGEVEWELPGSGSQQRWEDVV	Nestin	Nes	Q6P5H2		MEROPS	6642.0541	6661.0878	39.38	53.41	2.30	11.11	6.639	0.037
RPSTMLCAGYLAGGLDSCQGDSGGPLTCSEPGPRPREVLFGVTSWGDGCGEPGKPGVYTRVT	Serine protease 56	Prss56	F2YMG0		SignalP	6508.0191	6527.0477	38.22	52.51	1.57	15.34	6.634	0.037
STPTPTTTASSTASGSAPNPTTTVSSTASGSTPTLPTTASSSGSGSTPTLTTTESSTASGSSPTLTTTASSSA	Protein Gm9573	Gm9573	F7C950		SignalP	6651.0814	6670.0954	38.21	53.40	0.65	15.36	6.631	0.037
SDGSSTPARATVTLNVTDVNDN	Protein Pcdh9	Pcdh9	F8VPK8	A0A0A6YY09,A0A0A6YWY8,A0A0A6YWM0	SignalP	2215.0353	2234.0482	38.26	48.00	2.46	13.22	6.579	0.045
FKQDVFDFPACDVFTVEPGFDAALGQYLC	Nuclear pore membrane glycoprotein 210	Nup210	A0A0R4J1I6	Q9QY81	SignalP	3337.5100	3356.5521	38.87	53.46	7.12	16.52	6.536	0.045
VVFVVLVLVLTGSLVALAYLCVLPLLLRTY	Probable palmitoyltransferase ZDHHC16	Zdhhc16	Q9ESG8		SignalP	3297.9905	3317.0398	38.64	53.00	9.36	15.75	6.529	0.045
AEFQPLVEEPKNL	Serum albumin	Alb	P07724		MEROPS	1494.7718	1513.7940	32.87	66.55	2.54	15.89	6.524	0.045
AEFQPLVEEPKNLVKTNCDLYEKLGEYGFQNA	Serum albumin	Alb	P07724		MEROPS, SignalP	3724.8083	3743.8424	40.63	64.11	4.23	18.08	6.520	0.052
FSSLMNLEEKPAPAA	Apolipoprotein A-II	Apoa2	P09813		SignalP	1585.7810	1604.8040	31.21	70.02	2.90	15.31	6.505	0.052
LTSELTDERNTGESASQLLDAETA	Unconventional myosin-XVIIIa	Myo18a	Q9JMH9		MEROPS	2532.1827	2551.1922	25.56	52.97	3.51	15.23	6.495	0.052
KARENPSEEAQNLVEFTDE	Splicing factor 3A subunit 3	Sf3a3	Q9D554		MEROPS	2187.0080	2206.0304	38.60	47.30	1.84	14.08	6.462	0.059
DLVCSPVWTSRDRCCDLPSRRDEAKCPALPNACTCTQDSVGPPGPPGPAGGPGAKGPRGER	Collagen alpha-1(XII) chain	Col12a1	E9PX70	Q60847–5	SignalP	6581.0473	6600.0596	41.29	53.48	0.93	12.69	6.449	0.059
RGYEASVDSLTFGAVTGPDPSEEAGTKARFSLSDNVEEGSWSASVLDQQDNVLSLQLCTPAN	Protein-glutamine gamma-glutamyltransferase 2	Tgm2	P21981		MEROPS	6554.0731	6573.0724	38.80	52.57	2.92	17.55	6.381	0.067
HPPPTSREDKSPSEESTTTTSPESLSGSVPSSG	UV excision repair protein RAD23 homolog A	Rad23a	P54726		MEROPS	3336.5229	3355.5558	37.44	53.13	4.34	16.09	6.337	0.080
GRNQASAGSAPGAVLSQAMESTAVRPEETPRGLGDGLESSGTVQEPDAGGSSLEQDSQKQAEEKEQ	Scavenger receptor class F member 1	Scarf1	Q5ND28		SignalP	6678.1247	6697.1335	36.12	52.95	1.44	18.93	6.328	0.080
KSQPKKFCDYCKCWLADNRPSVEFHE	WW domain-binding protein 4	Wbp4	Q61048		MEROPS	3310.5110	3329.5493	39.73	53.65	6.01	14.28	6.327	0.080
TDGSFRCECPMGYNLDYTGVRCVDTDE	Fibrillin-2	Fbn2	Q61555		MEROPS	3198.2787	3217.2710	36.64	74.43	8.16	12.38	6.304	0.084
SSERVSGAEPAPGTMSKHRGKPSAACRCCVTYCEGESHLRSKSRAEMHTHPQWETHL	Isoform 3 of Interleukin-1 receptor accessory protein	Il1rap	Q61730–3		SignalP	6499.9797	6518.9949	38.98	52.98	0.48	14.82	6.285	0.084
LPFKNL	Cholesterol side-chain cleavage enzyme mitochondrial	Cyp11a1	Q9QZ82		MEROPS	712.4272	731.4462	52.82	109.68	0.87	17.99	6.244	0.094
NGLCVNSRGSFKCECPNGMTLDATGRLCLDLRLETCFLKYDDEECTLPLAGRHRMDA	Fibrillin-1	Fbn1	Q61554		MEROPS	6674.0571	6693.0873	39.87	53.46	1.76	13.49	6.228	0.094
NGRLTCTSRNRCNDQDTRTSYRLGDTWSKKDNRGNLLQCVCTGNGRGEWKCERHA	Fibronectin	Fn1	P11276		MEROPS	6596.0482	6615.0780	39.84	53.14	1.72	17.33	6.212	0.094
TVNPYTYPEDDYLPKFWVFFFKCSFSEFDCQLLENCQPNASLDLLPRHLFDPAMS	Protein Vmn2r62	Vmn2r62	K7 N712		SignalP	6690.0785	6709.0974	42.18	53.73	0.08	17.11	6.206	0.094
KSGVGGMGGYPGPAGPPGPPGPPGSS	Collagen alpha-1(III) chain	Col3a1	P08121		MEROPS	2200.0371	2219.0434	41.73	48.05	5.49	18.01	6.165	0.095
SSLDGLLTEHGPFLL	Lysosomal protective protein	Ctsa	P16675		MEROPS, SignalP	1579.8246	1598.8403	25.28	37.22	1.69	12.40	6.162	0.095
CSGSLVERRPCFSALTVD	Serum albumin	Alb	P07724		MEROPS	2034.9615	2053.9827	29.23	47.08	1.38	11.05	6.072	0.097
LVSARSVSPTTEMVSNESVDYRATFPEDQFPNSSQNGACRQVQYPLTDLSPLLTSGDSDLS	Hepatocyte growth factor receptor	Met	F8VQL0	P16056	SignalP	6645.1187	6664.1136	31.59	55.15	3.54	11.68	6.071	0.097
PFPTFSSTAVMAKETTAFEEGEGSTYTPSEGRLMTGSERVPGLETTPVGTSYPPGALTDQEVE	Versican core protein	Vcan	Q62059		MEROPS	6606.0893	6625.0931	41.40	52.72	2.22	14.43	6.067	0.097
KLLLAFSLLLVLLLFQEQL	Protein Vmn2r23	Vmn2r23	E9PXI5		SignalP	2195.3697	2214.3732	41.79	47.98	6.78	17.75	6.050	0.101
ELETSHLGKGCDRDTYSEKSLHRLCGAAAGTSELLPSPSSSFNWTVGLPTDNGHDSDQVFE	Calsyntenin-1	Clstn1	Q9EPL2		MEROPS, SignalP	6644.0520	6663.0569	38.66	52.84	2.03	11.98	6.032	0.101
QATTQPSTTAGTSTTTTTTTTAA	Ubiquilin-2	Ubqln2	Q9QZM0		MEROPS	2182.0237	2201.0355	39.22	47.21	3.03	13.97	6.025	0.101
PPASVVVGPVVVPR	Alpha-2-HS-glycoprotein	Ahsg	Q3UEK9	P29699	SignalP	1353.8132	1372.8346	32.85	63.44	2.21	11.64	5.985	0.101
EEAPQPALPFQPDSPTHFTP	Isoform 3 of Seizure protein 6	Sez6	Q7TSK2–3		SignalP	2187.0272	2206.0278	36.80	47.80	8.16	17.75	5.964	0.105
ATADAGSLSPRTCAALQKALDDDNDEKVSGSSDDLAEKMLLGSGLEQEEHADETAERGGGVPFD	DNA repair protein complementing XP-G cells homolog	Ercc5	P35689		MEROPS	6628.0364	6647.0762	38.70	52.58	3.22	17.40	5.952	0.105

^a^Precursor retention time

^b^Precursor drift time

^c^Precursor mass error in ppm

^d^Maximum product mass error in ppm

### MWCNT-induced circulating peptidome contributes to cardiovascular endothelial responses

**Fig. 3:**
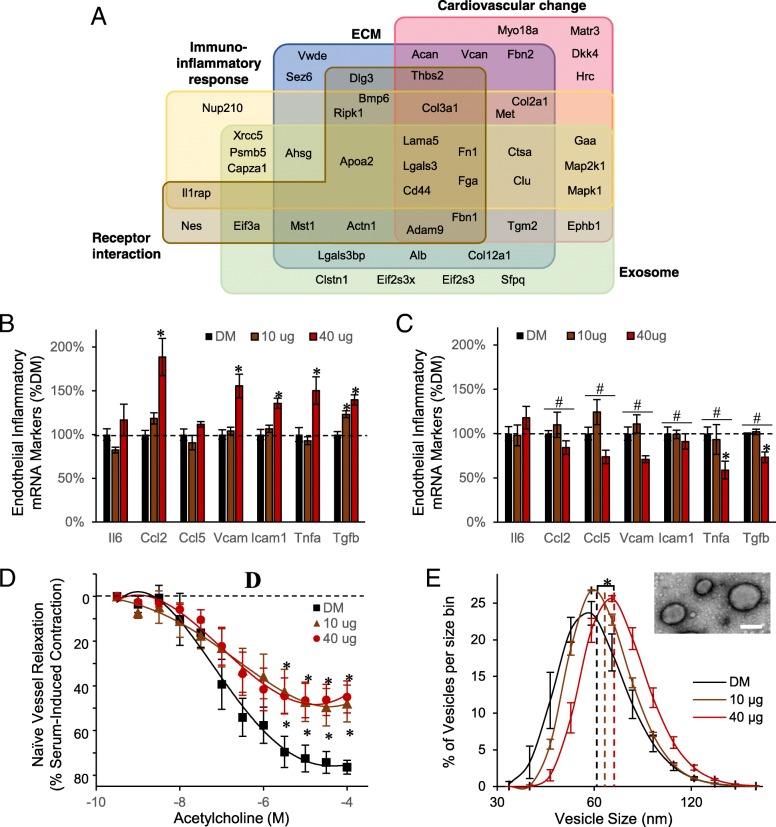
Functional relevance of the MWCNT-responsive peptidome. **a** Venn diagram reflecting functional associations among 73 identified peptides per ontology enrichment analysis. Groups denote the five most-enriched associations per biochemical, pathological, cellular and localization databases, with peptide-precursor protein symbols shown. Serum cumulative inflammatory potential assay results after treating endothelial cells for 4-h in vitro with **b** the enriched-peptide serum fraction and **c** after denaturing the peptide fraction. Presented as the mean ± SE, *n* = 6 serum peptide fractions from replicate mouse exposures per dose, ^#^*p* < 0.05 for effect of denaturing; *p < 0.05 for effect of MWCNT. **d** Acetylcholine induced vascular relaxation assessed ex vivo using naïve aortic rings treated with the enriched-peptide serum fraction, expressed as a percent recovery towards the pre-contraction tension. Data are presented as the mean ± SE, *n* = 6 serum peptide fractions from replicate mouse exposures per dose, *p < 0.05. **e** The hydrodynamic vesicle size distribution for an exosome-enriched size-exclusion serum fraction, plotted as the mean ± SE for bins sized between 30 and 180 nm, *n* = 3 replicate exosomal serum extracts from *n* = 6 replicate mouse exposures per dose. Dashed lines denote the curve mean, *p < 0.05. Inset electron micrograph of the serum exosomes; scale bar = 100 nm

Bioinformatic functional enrichment analysis was performed to inform on the pathobiological relevance of the circulating peptidomic response to MWCNT. Results classified into five enriched associations (Fig. 3a and Table 2). The largest group of 31 peptides were associated with an extracellular matrix localization and functionally to extracellular matrix (ECM) organization. With ECM reorganization being a principle factor in cardiovascular disease and inflammatory lesion development, it was also fitting that many of the ECM-related measures were among the 27 peptides enriched in association with an abnormal cardiovascular phenotype and altered cardiovascular proliferative biological processes. The results also included 18 peptides reflecting relevance to cell-surface receptor interactions denoting signaling ligands within the peptidome. Peptide-receptor interactions are involved in numerous biological processes, including immune and associated inflammatory signaling, with 22 associated peptides. Lastly, 29 peptides were derived from known exosomal localized proteins, suggesting exosome involvement in systemic MWCNT responses. Collectively, the peptidomic fraction biochemically paralleled our prior published inflammatory and vascular deficit outcomes induced in vivo *and* ex vivo using the present MWCNT exposure model [14, 23, 25].

**Table 2:** Enriched cellular, molecular and phenotypes associated with the MWCNT-induced peptidome

Category	ID	Name	q-value	#Hits
Extracellular matrix related				
CellComp^a^	GO:0005615	Extracellular space	4.15E-08	23
Pathway^b^	M5889	Genes encoding ECM and ECM-associated proteins	5.048E-6	20
BioProcess^c^	GO:0030198	Extracellular matrix organization	3.174E-09	15
Cardiovascular change related				
MPhenotype^d^	MP:0002127	Abnormal cardiovascular system morphology	0.01783	24
BioProcess^c^	GO:0072358	Cardiovascular system development	1.503E-5	18
Immuno-inflammatory response related				
BioProcess^c^	GO:0006955	Immune response	0.02223	14
Pathway^b^	1269203	Innate immune system	0.007017	16
Receptor binding related				
MolFun^e^	GO:0005102	Receptor binding	0.002014	17
Exosome related				
CellComp^a^	GO:0070062	Extracellular exosome	4.43E-05	29

Enrichment assessed in association with the following ontology database categories:

^a^cellular component

^b^molecular pathway

^c^biological process

^d^mouse phenotype

^e^molecular function

After establishing its pathobiological relevance, we then sought to assess the bioactive potential of the serum peptidomic fraction. The enriched peptide fraction was first assessed for its inflammatory potential on primary murine vascular endothelial cells, as previously reported for whole serum [14]. The assay assessed ex vivo endothelial induction across a battery of adhesion molecule and cytokine gene products linked with inflammatory-mediated immune responses. Naïve endothelial cells were treated in culture with the serum peptide fractions of MWCNT-exposed or DM control animals. As observed previously using whole serum from exposed animals [14], the peptide-fraction alone induced significant increases in canonical inflammatory markers *Ccl2*, *Vcam1*, *Icam1*, and *Tnfa*, principally at the 40 μg MWCNT dose relative to DM control (Fig. 3b). The magnitude of response was 189% (*p* < 0.001), 156% (*p* < 0.001), 136% (*p* < 0.001), and 150% (*p* < 0.033) of DM control levels for *Ccl2*, *Vcam1*, *Icam1*, and *Tnfa*, respectively. In contrast, *Ccl5* showed no response to treatment. *Tgfb* and *Il6* were additionally assessed in this study, relevant to the MWCNT peptidome’s enriched association with immune response processes. Application of the serum peptide fraction induced significant dose-dependent increases in *Tgfb* expression: 123%, *p* < 0.001 at 10 μg; 140%, *p* < 0.001 at 40 μg. In contrast, *Il6* levels were absent significant effects of treatment. To support that the inflammatory response was driven by peptide components within the enriched fraction, an aliquot was denatured by reduction and alkylation to disrupt peptide structure, buffer exchanged, and applied to naïve cerebrovascular endothelial cells. Notably, denaturing significantly reversed the peptide fraction’s prior inflammatory potential: *Ccl2* (40 μg, *p* < 0.001), *Ccl5* (40 μg, *p* = 0.004), *Vcam* (40 μg, *p* < 0.001), *Icam1* (40 μg, *p* < 0.001), *Tnfa* (40 μg, *p* < 0.001), and *Tgfb* (10 μg, *p* = 0.002; 40 μg, *p* < 0.001) (Fig. 3c).

Next, the serum peptide fraction was assessed for its potential to induce vascular dysfunction as previously tested for whole serum using myography [25]. Naïve aortic rings treated with the serum peptide fraction from 10 μg and 40 μg dosed animals exhibited significant dysfunction (F = 31.2, *p* < 0.001, main effect of MWCNT exposure) in acetylcholine-mediated dilation (Fig. 3d). The effect was dose-independent, reaching only 57% the dilation achieved with DM control at either MWCNT dose. As a percent of DM control, the peptide fraction induced the same magnitude deficit as whole serum treatment, which reach just 56% of DM control relaxation at the 40 μg dose [25]. However, the response to the peptide fraction lacked an added deficit previously reported with whole serum at the 10 μg dose, which dilated to only 29% that reached with DM control serum.

### MWCNT-responsive exosome-associated peptides coincide with serum exosomal shifts

As the MWCNT-responsive peptidome was significantly enriched in association with exosomes, we sought to next test an association of this change with a parallel increase in serum exosomes. An enriched exosomal fraction was purified by ultrafiltration followed by size-exclusion chromatography. The vesicle size distribution fell between 40 and 160 nm (Fig. 3e) and excluded serum proteins that appear in later fractions between 6 and 20 nm. The measured vesicle distribution matched precisely that accepted for exosomes [49]. MWCNT exposure induced a dose-dependent shift to larger-sized serum-exosomal vesicles, reaching significance for the 40 μg dose (18.3% increase, *p* < 0.001): DM, 60.4 ± 1.0 nm; 10 μg, 64.7 ± 0.9 nm; 71.4 ± 0.4 nm. Furthermore, the area under the hydrodynamic-size curve increased with MWCNT exposure, relative to DM control, purporting a greater number of exosome-sized serum vesicles, which reach significance at the 40 μg dose (17.5% increase, *p* = 0.0284): DM, 924 ± 40.6; 10 μg, 974 ± 8.3; 40 μg, 1086 ± 26.1. The enriched vesicles were further assessed by transmission electron microscopy (Fig. 3e), with their size and morphology consistent with that previously published for exosomes [50].

### Identified thrombospondin fragment relates peptidome relevance to vascular dysfunction

**Fig. 4:**
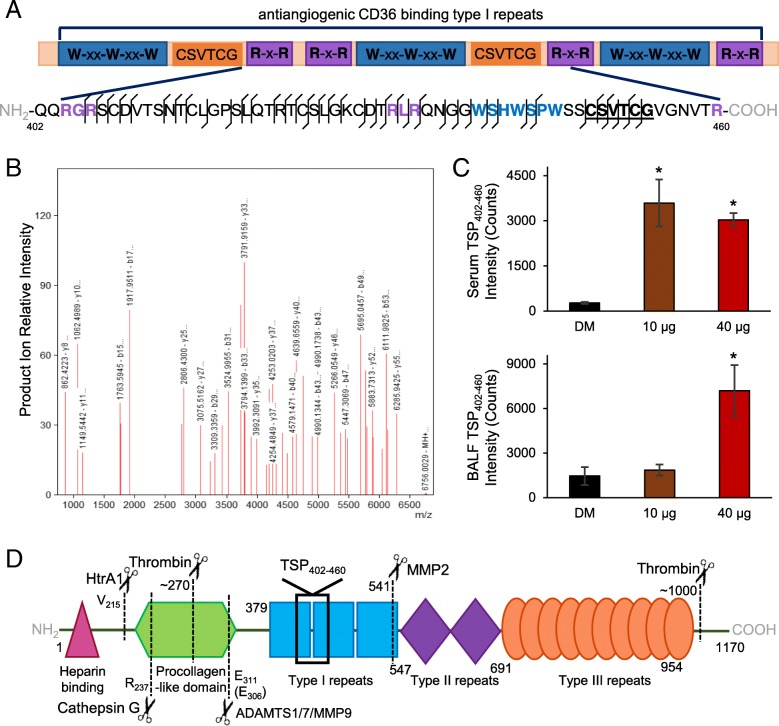
MWCNT-responsive thrombospondin fragment identified with CD36-modulatory motifs. **a** Among the identified peptides was a 59-mer thrombospondin fragment overlaping the second type-1 repeat domain (TSP_402–460_). Three established CD36 binding and modulatory motifs are color-coded and shown in bold within the peptide sequence. Matched mass spectral ion fragmentation sites are shown along the 59-mer sequence with a line angled to the left denoting a b-ion (left-to-right) a line angled to the right denoting a y-ion (right-to-left). **b** The TSP_402–460_ peptide’s corresponding fragment ion mass spectrum with peaks labeled for their reduced mass (MH+) and b/y-ion designation. **c** Mass spectral ion intensity for the TSP_402–460_ peptide plotted as mean ± SE, *n* = 6 matched serum and BALF specimens from replicate mouse exposures per dose, *p < 0.05. **d** Proteolytic processing map for known thrombospondin cleavage sites with the protease and site given relative to functional domains

The induction of endothelial-dependent vasodilatory deficits by serum factors from MWCNT exposed animals has recently been independently confirmed [25, 26]. The two studies offer complementary mechanistic insight demonstrating the co-dependence between MMP9 activity and TSP-mediated CD36 signaling; yet, a molecular interconnection was not readily apparent. Included within the MWCNT-responsive peptidome reported here was a 59-mer fragment from a TSP2 type-1 domain between residues 402–460. Within TSP type-1 domains are three motifs that enable CD36 binding and modulation: R-x-R, W-xx-W-xx-W and CSVTCG motifs [51, 52]. The identified TSP_402–460_ peptide contains all three motifs (Fig. 4a), which was well characterized by the product ion spectra (Fig. 4b). Significant increases in the TSP_402–460_ peptide were measured dose-independently within serum at the 10 μg (*p* = 0.002) and 40 μg (*p* < 0.001) doses, but dose-dependently in BALF at the 40 μg dose (*p* = 0.002) relative to DM control (Fig. 4c). The average serum TSP_402–460_ peptide concentration was then approximated at 24 nM and 20 nM in serum from 10 μg and 40 μg MWCNT-dosed animals, respectively, using the mean mass spectrometer response to a known peptide concentration. The similar concentration between the two MWCNT doses paralleled the comparable vascular deficit induced by the serum peptide fraction per myography (Fig. 3d). Furthermore, the increased serum amount of the 59-mer peptide at the two MWCNT doses closely profiled MMP9 protein levels within lung tissue (Fig. 2b). Yet known MMP9 cleavage of TSP between residues 306–307 sits well away from the TSP_402–460_ peptide identified here (Fig. 4d), suggesting additional tandem cleavage events by other proteases. TSP is well known to be proteolyzed by other matrix-related enzymes such as MMPs 2 and 14, ADAMTSs 1 and 7, and cathepsin G [53–56]. Moreover, TSP is proteolyzed by immune-cell released and circulating enzymes, for example, elastases, thrombin, plasmin, and HtrA1 [56–60]. The complement of established cleavage sites for these proteases (Fig. 4d), however, are likewise nonspecific to the sequence of the TSP_402–460_ peptide. Recognizing that the full complement of TSP cleavage events, particularly with sequential processing by multiple proteases, cannot be known, we then looked to site prediction informatics. Using the feature-based tool PROSPER [61], we resolved that the c-terminal side of the TSP_402–460_ peptide is a probable neutrophil elastase cleavage site (cleavage probability score 1.11 > 0.8 cutoff for positive prediction at an 82.9% accuracy for elastase-2). While the full complement of proteolytic processing needed to yield this peptide is expectedly complex, its retained CD36 ligand characteristics exemplify the bioactive potential of the MWCNT-induced circulating peptidome.

### Peptide-mediated model of systemic effects following pulmonary nanoparticle exposure

**Fig. 5:**
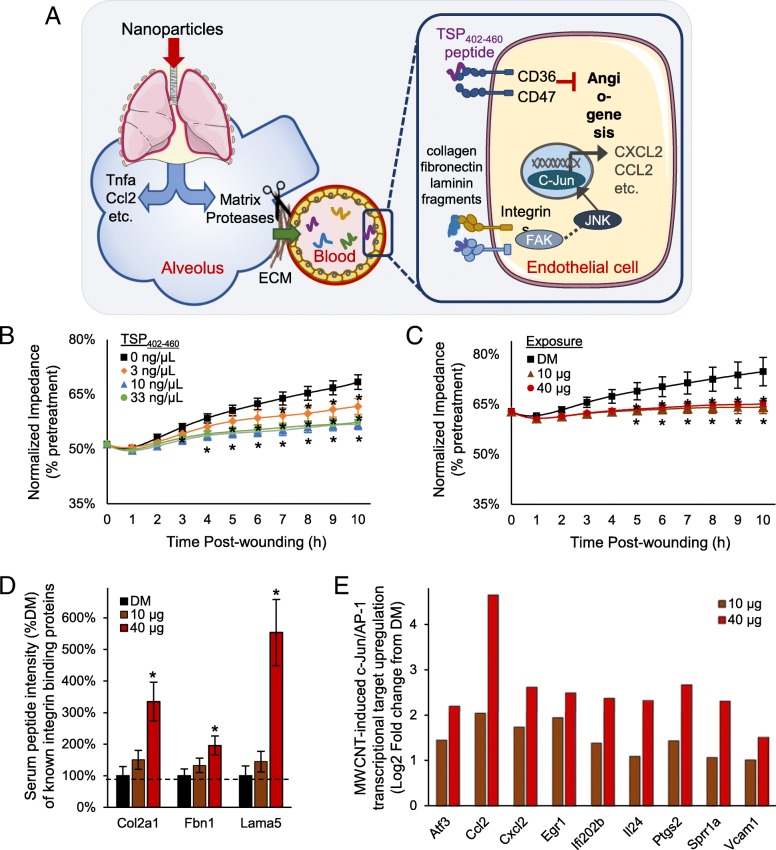
Peptide-mediated model of systemic bioactivation following pulmonary nanoparticle exposure. Results from this study support a proposed paradigm (**a**) by which nanoparticles in the lung activate matrix proteases, with a diversity of generated peptide products released into circulation with some acting as cell-surface receptor ligands that drive systemic vascular dysfunction and inflammation. The model depicts peptides products of thrombospondin and integrin-ligand proteins from the MWCNT-responsive peptidome triggering CD36 and integrin receptor signaling with downstream anti-angiogenic and inflammatory marker outcomes. **b** Following synthesis of the identified MWCNT-responsive TSP_402–460_ peptide, its anti-angiogenic properties were assessed using an electronic wound-healing assay after a 4-h treatment. **c** Likewise, the electronic wound-healing assay was used to assess the anti-angiogenic properties after a 4-h treatment with the serum peptide fraction from *n* = 6 MWCNT treated animals per dose. Electrical impedance values were plotted per hour for 10 h following wounding, with data centered and normalized as a percentage of pretreatment impedance and given as mean ± SE, *n* = 6 in vitro replicates, *p < 0.05. **d** Peptides identified from known integrin-binding proteins were dose-dependently increased, mass spectral intensity normalized as a % of DM vehicle control presented as mean ± SE, *n* = 6 replicate exposures per dose, *p < 0.05. **e** c-Jun transcriptional targets upregualted in endothelial cells treated 4-h in vitro with serum collected 4-h after MWCNT or DM control exposures. Mean log2 fold change results plotted, *n* = 4 replicate mouse exposures per dose, *p* < 0.01

Cumulatively, findings here establish a complex serum peptidomic response acutely following exposure to MWCNT that conveys bioactivity. Included among the responsive factors are peptide fragments related to abnormal cardiovascular and inflammatory responses, exemplified by the identification of a TSP_402–460_ peptide with CD36 binding motifs. Results here support consideration of a new peptide-mediated model of systemic effects (Fig. 5a) following nanoparticle exposure. Proposed is that a diverse assortment of matrix remodeling proteases activated following exposure induce peptide byproducts into circulation where they may be further modified yielding an expanded serum peptidomic complexity. The peptidomic response includes cell-surface receptor binding capacity that promotes extra-pulmonary effects. To validate the potential of this model, we synthesized the TSP_402–460_ peptide and applied it in a CD36/CD47-mediated endothelial cell in vitro wound healing angiogenesis assay (Fig. 5b). Addition of the TSP_402–460_ peptide into the culture media produced a significant anti-angiogenic effect (F = 43.5, *p* < 0.001), impairing cell regrowth as measured by recovered membrane impedance. Relative to vehicle control, a 6% deficit reestablishing baseline impedance 10 h after wounding was observed at a 3 ng/μL dose (*p* = 0.002), a 22 nM concentration in media similar to that estimated in the serum of exposed animals. A 3-fold or greater increase in TSP_402–460_ concentration saturated the deficit at 12% (*p* < 0.001). In comparison, the serum peptide fraction from MWCNT exposed animals also significantly inhibited (F = 32.7, *p* < 0.001, main effect of dose) endothelial cell regrowth out to 10 h after wounding (Fig. 5c). The induced deficit recovering baseline impedance was 9.5% (*p* < 0.001) relative to DM control, which fell between that induced at 3 and 10 ng/μL TSP_402–460_ peptide. These data establish the bioactive nature of the circulating TSP_402–460_ peptide that, at least in part, accounts for the anti-angiogenic capacity of the serum peptide fraction.

Looking beyond the TSP peptide, the MWCNT-responsive peptidome was enriched with other fragments of receptor-binding proteins (Fig. 3a). A number of them were derived from known integrin binding collagens, fibronectin and laminins, offering additional potential in mediating observed bioactivity. The MWCNT-response among these peptides was dose-dependent (Fig. 5d) similar to findings from the serum cumulative inflammatory potential assay (Fig. 3b), which contrasted with the dose-independent increase in the TSP_402–460_ peptide and CD-36 dependent myography (Fig. 3d) and wound healing (Fig. 5c) findings. The MWCNT-responsive peptidome was also significantly enriched in association with interactions upstream of c-Jun transcription (FDR 0.0127) and integrins are known to induce c-Jun-mediated inflammatory factor transcription. Supporting the integrin-ligand potential of the MWCNT-responsive peptidome, in vitro treated endothelial cells exhibited significant dose-dependent upregulation of numerous c-Jun transcriptional products (Fig. 5e) relative to DM control. Together, results here implicate integrin-ligand functionality of the MWCNT-responsive peptidome as an additional part of a broader combinatorial molecular response mediating systemic bioactivity after nanoparticle exposure.

## Discussion

The release of proinflammatory factors from the lung into the circulation, so-called “systemic spill-over”, has been posited as a causal link between respirable particle exposure and systemic toxicity, in lieu of, or in addition to particle translocation [6]. Yet uncertainty exists as to the molecular nature and diversity of what is released and how bioactivity is induced. The present study establishes the paradigm that proteolytic byproduct peptides are released from the lung as circulating mediators and potential biomarkers of exposure and/or extra-pulmonary health consequences. Peptide isolation by size fractionation and solid-phase extraction permitted the selective assessment of a peptidomic shift within the blood following MWCNT exposure, validated by unbiased liquid chromatography data-independent mass spectrometry to exclude hydrophobic lipid moieties and proteinaceous material above 7000 Da. The enriched peptide fraction conveyed inflammatory potential analogous to that previously demonstrated with whole serum from MWCNT-exposed animals when assessed ex vivo on naïve endothelial cells, despite mass-exclusion of typical cytokine/chemokine mediators. The serum peptide fraction’s inflammatory potential, however, was reversed when pretreated to denature the peptide content, confirming peptide involvement. The serum peptide fraction furthermore triggered vasodilatory and endothelial angiogenesis deficits in naïve vessels and cells ex vivo akin to that induced with whole serum. Collectively, these outcomes establish that the peptide fraction alone can convey much of the systemic bioactivity previously reported with full serum collected at the same 4 h time point after MWCNT exposure using the same model [14, 23, 25]. Moreover, the use of unbiased data-independent tandem mass spectrometry enabled partial identification of the MWCNT-responsive peptidome with a focus on correlated responses in the circulation and lung lavage to direct informatic inquiry towards biomolecular relationships with MWCNT-induced pulmonary pathobiology. The identified peptides were replete with fragments of known cell-surface receptor ligands, illustrated most notably by a peptide from the type-1 repeat domain of TSP. Containing binding and modulating motifs involved in promoting CD36-dependent endothelial dysfunction, this peptide exemplified a mechanistic role for circulating peptides triggering cell-surface receptor mediated systemic effects.

Nanoparticle exposure has previously been associated with protease activation [41]. Here we assert involvement of a broad array of differentially regulated MMPs, ADAMs and ADAMTS and associated metalloprotease inhibitors (TIMPs) in reaction to MWCNT pulmonary exposure. MWCNT can also promote lysosomal protease excretion [48], with several cathepsins involved in ECM remodeling found upregulated in the lung after MWCNT exposure, enhancing the potential diversity of proteolytic processes involved in establishing the MWCNT-responsive peptidome. Sequential multi-protease processing helps to explicate the wide diversity of peptide products discovered responsive to MWCNT, with significant correlation between lung lavage and serum fluid compartments denoting a pulmonary source for part of the circulating peptidomic response. TSP, for example, is an established substrate for at least five matrix metalloproteases and one cathepsin (MMPs 2, 9 and 14; ADAMTSs 1 and 7; cathepsin-G) [53–56]. TSP is also a known substrate for circulating proteases such as thrombin and Htra1 that also influence ECM reorganization [58, 59]. While a full decoding of sequential proteolytic processing of TSP falls beyond the scope of the present work, the identified TSP_402–460_ peptide points to additional, as yet unknown cleavage events. Site prediction software suggests that the c-terminal site represented by this peptide is a probable neutrophil elastase cleavage point, supported by the significant acute influx in lung neurophils using this model [23] and TSP as a known substrate of neutrophil elastase (exact sites unfound) [56, 60]. Altogether, the peptidomic response revealed by these studies represents a post-exposure molecular complexity as yet unappreciated, demarking an equally complex pathobiological response, with substantial diagnostic potential.

Identification of the MWCNT-responsive peptides revealed here is challenged by the stochastic nature of their endogenous generation. Unlike bottom-up proteomic mass spectrometry that involves in-tube digestion with a positionally-restrictive enzyme like trypsin, unknown endogenous byproduct peptides have no stipulated restraints on their beginning or end residues, obfuscating proteomic search engines. To alleviate some of this burden we employed target databases to significantly narrow the sequence search space [62]. We further utilized FDR-controlled peptide match scores rather than protein-level scoring, which is the default of most search engines that assume multiple peptides should match to a single protein in a confident identification. Focused here on peptides measured commonly between serum and BALF to provide selective relevance to pulmonary pathobiology, we were able to identify 73 in connection with known matrix protease substrates and signaling motifs. As a representative sampling of the peptide fraction, there was significant enrichment in matrikine-like fragments from ECM proteins with receptor-ligand functionality. For example, peptides of laminin 5 (Lama5), fibronectin 1 (Fn1) and collagen 3a (Col3a1) were identified, all ECM proteins known to interact with integrin surface receptors. Furthermore, the MWCNT-responsive peptidome was significantly associated with c-Jun signal transduction. Integrin signaling activates a variety of cellular processes, to include c-Jun n-terminal kinase (JNK) mediated regulation of cell proliferation, migration, and cytokine/chemokine production. ECM-derived peptides acting as circulating integrin ligands can moreover explain dose-dependent endothelial cytokine/chemokine induction observed ex vivo when treating with serum from MWCNT-exposed animals [14, 25]. In affirmation, microarray analysis of ex vivo treated naïve endothelial cells showed significant upregulation of pathway targets downstream of JNK, reflecting inflammatory marker production and cell stress responses.

The serum peptide fraction alone induced much of the proinflammatory marker response previously reported with whole serum ex vivo treatment [14, 23, 25]. Canonical inflammatory markers *Ccl2*, *Vcam1*, *Icam1*, and *Tnfa* were all significantly elevated in a dose-dependent manner. Furthermore, the magnitude of response observed here was on-par with that produced by whole serum. Given that the sample fractionation process excluded larger-mass cytokines and chemokines, bioactivity induced by the serum peptide fraction must reflect the presence of alternative proinflammatory ligands such as the dose-dependently increased fragments of integrin-binding proteins *Col2a1*, *Fbn1*, and *Lama5* or other cell-surface ligands such as gelactin 3 (Lgals3) or VG-1-related protein (Bmp6), with future studies warranted to clarify details on specific peptide involvement. The ex vivo endothelial proinflammatory response to the peptide fraction, however, was not entirely the same, lacking the robust *Ccl5* upregulation induced with whole serum. We ascertained that the enriched peptide fraction lacked some components of the circulating bioactive profile. It is likely that larger proteolytic fragments, as well as other secreted mediators, were excluded from the peptide fraction. Non-peptide factors also surely contribute to the response diversity across doses and xenobiotic exposures. Yet a majority of the dose-dependent inflammatory response previously reported were reproduced with the serum peptide fraction studied here, substantiating proteolytic product involvement as an indirect mediator conveying a MWCNT response across the lung-blood barrier and acting as cell-surface receptor ligands to promote systemic effects (Fig. 5a). The peptidomic findings here thus help expound on the molecular “systemic spill-over” response as more substantially diverse than previously appreciated.

The identified TSP_402–160_ fragment was studied here in greater detail as a prototypic example of the MWCNT-responsive peptidome’s contribution to cell-surface mediated systemic effects. The 59-mer spans the second of three TSP type-1 repeats, which has been reported as the principal CD36 binding site, having the positively charged W-xx-W-xx-W and R-x-R motifs needed to interact with the negatively charged CD36 CLESH domain [51, 63]. The fragment also contains the CSVTCG motif found to modulate CD36 activation [52]. Studies show that synthetic peptides derived from TSP type-1 repeat domains exhibit anti-angiogenic properties in a CD36-dependent manner [64], and synthetic peptides as short as 18 amino acids containing the W-xx-W-xx-W motif [62] are potent inhibitors of cell proliferation and angiogenesis [65]. Thus the TSP_402–460_ peptide was synthesized and tested for anti-angiogenic properties using a high-precision electrical wound-healing assay. Testing the TSP_402–460_ peptide at a comparable nanomolar concentration in vitro induced a vascular regrowth deficit on par with the serum peptide fraction from MWCNT-exposed animals (Fig. 5c), confirming its bioactivity. We previously published that MWCNT-diminished vasodilatory responses were CD36-receptor mediated, with knockout animals exhibiting a muted response [25]. Mandler et al. also found that serum from TSP knockout animals treated with Mitsui-7 MWCNT lacked the ability to induce vasodilatory deficits in naïve aortas [26, 66]. They also showed that knocking out CD47 also offered vascular protection in ex vivo assays with serum from MWCNT exposed animals, which fits the dependence of TSP-CD36 driven nitric oxide responses on proximal localization of CD47 without the need for direct TSP-CD47 binding [67]. Yet circulating TSP increased by only 50% 4 h following in vivo MWCNT exposure, which is unlikely to dramatically perturb endothelial homeostasis [14]. Furthermore, testing MWCNT-induced vasodilatory dysfunction ex vivo was also largely abolished using serum from MMP9-deficient mice [25], demonstrating a co-dependence of these effects on MMP9 processing. Missing was a factor(s) interlinking TSP, CD36 and MMP9. The identified TSP_402–460_ fragment peptide offers a unifying mediator of CD36-related systemic pathobiology, increased in serum by over an order in magnitude, and well in excess of the modest 0.5-fold MWCNT-induced increase in circulating TSP protein [14].

Notably, results from this study emphasize that the peripheral molecular response to nanoparticle exposure is considerably more diverse than previously appreciated. It then fits that systemic bioactivity occurs as a combinatorial response to several circulating factors. The peptide-mediated model in Fig. 5a proposes the involvement of multiple ligand-receptor triggered events. We surmise that this explains the produced dose-independent myography and wound-healing results that were consistent with a dose-independent magnitude of TSP_402–460_ peptide in circulation, which stands apart from the dose-dependent endothelial induction of pro-inflammatory cytokines and chemokines that are not downstream of CD36-receptor signaling. Inflammatory marker production is, however, downstream of integrin-mediated c-Jun transcriptional activity. The MWCNT-responsive peptidome was enriched in peptides from receptor binding proteins, particularly integrin ligands upstream of c-Jun. The identified fragments of known integrin binding ligands exhibited dose-dependent increases, warranting follow-up investigation as to the combinatorial bioactivity across the responsive peptidome.

Unexpectedly, we found an enriched association between the MWCNT-responsive peptidome and exosomal protein fragments. Exosomes play a pivotal role in cellular communication by delivering bioactive molecules such as mRNA, miRNA, proteins and perhaps peptides to distant tissues. Yet exosomal involvement in response to inhaled xenobiotics remains understudied; though, it was recently published that bronchial epithelial cells release exosomes when challenged with cigarette smoke extract [68]. It turns out that very few proteins, mainly surfactant-related, are specific enough to demark a vesicular lung origin. While the data here cannot affirm lung specificity, they do suggest it, with 28 of 29 exosome-associated peptides derived from proteins of known lung expression and 77.3% with a higher than average abundance (Tissue Atlas, www.proteinatlas.org). Presently, we found an increase in the size and abundance of serum exosomes following MWCNT exposure. An increase in diameter infers a greater volumetric increase in vesicle capacity, suggesting a quantitative shift in exosomal cargo as seen under other pathological conditions [69–71]. These findings demonstrate that MWCNT exposure alters the serum exosome population, implicating their potential involvement in systemic pathobiology. Exosomes also carry matrix proteases and thus may play a further role in peptidome processing [71]. Follow-up studies are needed to determine whether exosome-related peptides found here reflect induced surface protein cleavage or rather a shift in peptide exosomal packaging with separate bioactive capacity.

Further studies are needed to address additional questions raised from the initial findings here, to include assessing ligand functions of other identified peptides and determining selectivity within the peptidomic response across different pulmonary exposures – whether different carbon nanomaterials or other types of nanoparticles. Different exposures are likely to exhibit a diversity of proteolytic events and a correspondingly distinctive peptidomic signature that is consistent with differences in pulmonary injury. Modulation of MWCNT dose was alone sufficient to shift the complexity of the peptidomic response, and time after insult is yet another key variable that needs to be examined. Perhaps peptidomic distinctions between materials would be more evident at later time points following what may be a more generalized acute-phase response studied here. It should also be noted that 77% of the MWCNT-responsive peptidome common between serum and BALF was not readily identified, and that the added peptidomic complexity observed exclusively in the circulation, relative to BALF, remains to be examined. As discussed earlier, endogenous peptides are particularly difficult to identify due to the vast sequence-search space involved. We used smaller targeted databases to facilitate identification; however, products outside those focused databases go unidentified. Informatic approaches to improve search selectivity are also needed to address identification of post-translational modified peptides. Blood polypeptides are often glycosylated, a particularly diverse modification that exacerbates the sequence search space issue and further complicates comprehensive identification. Ultimately, results here establish existence of a diverse and bioactive circulating peptidomic response following pulmonary nanoparticle insult, providing foundation for more through characterization of its mechanistic role in driving systemic pathobiology.

## Conclusions

Findings here substantiate proteolytic peptide involvement in mediating systemic consequences of nanoparticle exposure. A wide variety of inhaled xenobiotics upregulate matrix proteases and produce various systemic effects. Agents from titanium dioxide to diesel exhaust have been shown to induce extra-pulmonary inflammation and cardiovascular impairments much as reported here for MWCNT [27, 72–74]. Indeed, many insoluble metal and engineered nanoparticles (Au, Ag, titanium dioxide, ultrafine carbon and other CNTs, etc.) induce pulmonary pathology that may be accompanied by proteolytic generation and release of bioactive peptides. Once in circulation, fragments such as the TSP_402–460_ peptide or those from integrin ligands induce cell-surface receptor mediated systemic stress and inflammation, driving particulate exposure’s adjuvant role in promoting everything from cardiovascular to neurodegenerative diseases. Furthermore, the revealed existence of a pathological peptidomic response to nanoparticle exposure represents a highly diverse and dose-dependent molecular source of putative biomarkers that may provide greater specificity than previously available [9]. More thorough characterization of circulatory factors is warranted to assess the role of peptide byproducts mediating systemic disease following pulmonary insult to a broader array of occupational and environmental xenobiotic agents.

## Abbreviations

BALF: Bronchoalveolar lavage fluid
*Ccl2*: C-C motif chemokine ligand 2
*Ccl5*: C-C motif chemokine ligand 5
DM: Dispersion media
ECM: Extracellular matrix
FDR: False discovery rate
Il6: Interleukin 6
MMP: Matrix metalloproteinase
MWCNT: Multi-walled carbon nanotubes
*Tgfb*: Transforming growth factor beta
*Tnfa*: Tumor necrosis factor alpha
TSP: Thrombospondin
*Vcam1*: Vascular cell adhesion molecule 1
